# Switching from robotic-assisted extended transabdominal preperitoneal (eTAPP) to totally extraperitoneal (eTEP) hernia repair for umbilical and epigastric hernias

**DOI:** 10.1038/s41598-024-52165-6

**Published:** 2024-01-20

**Authors:** Ramon Pini, Francesco Mongelli, Fabiano Iaquinandi, Paolo Gaffuri, Marco Previsdomini, Agnese Cianfarani, Davide La Regina

**Affiliations:** 1 Department of Surgery, Bellinzona e Valli Regional Hospital, EOC, Via Gallino 12, 6500 Bellinzona, Switzerland; 2 Faculty of Biomedical Sciences, Università Della Svizzera Italiana, 6500 Lugano, Switzerland; 3 Intensive Care Unit, Bellinzona e Valli Regional Hospital, EOC, 6500 Bellinzona, Switzerland

**Keywords:** Gastrointestinal diseases, Gastrointestinal system

## Abstract

Our study aimed to assess the safety and effectiveness of the robotic-assisted extended totally extraperitoneal (eTEP) repair compared to transabdominal preperitoneal (eTAPP) repair with a suprapubic trocar insertion to treat umbilical and epigastric hernias. On a prospectively maintained database, we identified patients who underwent either eTEP or eTAPP for treating umbilical and epigastric hernias. During the study period, 53 patients were included, 32 in the eTEP group and 21 in the eTAPP group. The mean age was 59.0 ± 13.9 years, 45 patients (84.9%) were male, and the mean BMI was 28.0 ± 5.9 kg/m^2^. Most hernias were umbilical (81.1%) and primary (83.0%). The operative time for eTEP was slightly shorter than for eTAPP (106 ± 43 min vs. 126 ± 74 min, p = 0.232). Postoperatively, only one case of bleeding and one seroma were recorded. No complication occurred during a mean follow-up of 11.3 ± 6.4 months in the eTEP group and 20.5 ± 9.7 months in the eTAPP group. In conclusion, our study showed that the eTEP with suprapubic approach was safe and feasible in the treatment of epigastric and umbilical hernias. According to our experience, shorter operative time, integrity of the posterior layers and increased overlap size are the main surgical reasons of switching from eTAPP to eTEP.

## Introduction

Since its first description by LeBlanc and Booth in 1993, laparoscopic intra-peritoneal onlay mesh (IPOM) has rapidly established itself as a safe alternative to open ventral hernia repair^1^. However, over the past two decades, many attempts have been made to overcome the disadvantages of intraperitoneal mesh placement and fixation, such as intestinal erosion, obstruction, fistulae, and postoperative pain^2–4^. Those efforts led to the development of diverse minimally invasive techniques^5–7^, furtherly facilitated by the widespread use of robotic platforms which, according to numerous authors, provide technical advantages in dissection, components separation, fascial defect closure and mesh fixation^8,9^.

Over the past years, a few studies reporting on the safety and feasibility of minimally invasive treatment of epigastric hernias with extended transabdominal preperitoneal technique (eTAPP) have been published^7,9,10^. In a previous study, we described our experience on robot-assisted, transabdominal repair (eTAPP) of epigastric hernias with suprapubic approach^7^.

In 2012, Daes et al. described the enhanced view totally extraperitonal (eTEP) for difficult inguinal hernia repair^11^. Later, Belyansky et al. presented their experience of eTEP in ventral hernia repair, using laparoscopic and robotic-assisted approaches^12,13^.

In the recent years, keeping the suprapubic approach, we have switched from robotic eTAPP to eTEP in the treatment of umbilical and epigastric hernias.

The aim of our study was to assess the safety and feasibility of the robotic-assisted eTEP with suprapubic trocar insertion in the treatment of umbilical and epigastric hernias. In addition, we wanted to share our experience on the switching from eTAPP to eTEP, clarifying why we chose this path.

## Material and methods

### Study design and patient selection

Our institution is a specialized referral center for minimally invasive abdominal wall surgery. We retrospectively selected from a prospectively maintained database patients who underwent robotic-assisted umbilical and epigastric hernia repair with suprapubic approach, either eTEP or eTAPP. We included both primary and incisional hernias. The primary endpoint was to assess the safety and feasibility of the robotic-assisted eTEP compared to the eTAPP technique.

The study was approved by the local ethic committee (Comitato Etico Cantonale Ticino, 2019-01132 CE 3495), and informed consent was obtained from included patients. This research was conducted in accordance with current international regulations. Strengthening the reporting of observational studies in epidemiology (STROBE) guidelines were followed^14^.

Patients were identified on a prospectively maintained and audited electronic dataset specifically designed for patients undergoing hernia surgery (www.herniamed.de). We retrieved data on age, sex, height, weight, body mass index (BMI), presence of comorbidities (smoking status, chronic obstructive pulmonary disease, diabetes, American Society of Anesthesia (ASA) score, previous abdominal surgery, type of surgical approach (i.e., eTEP or eTAPP), type of hernia, hernia defect size, type and dimension of implanted mesh, intraoperative complications and postoperative complications according to the Clavien-Dindo classification^15^, hospital stay, follow-up visits. The follow-up included a clinical assessment 30 days after surgery and subsequently annually for up to five years post the hernia operation. The choice between eTAPP and eTEP was based on the surgeon’s experience. We started performing eTAPP for umbilical and epigastric hernias, and then we decided to switch to eTEP as some advantages might be offered.

### Surgical technique of robotic-assisted eTEP with suprapubic approach

**Figure 1:**
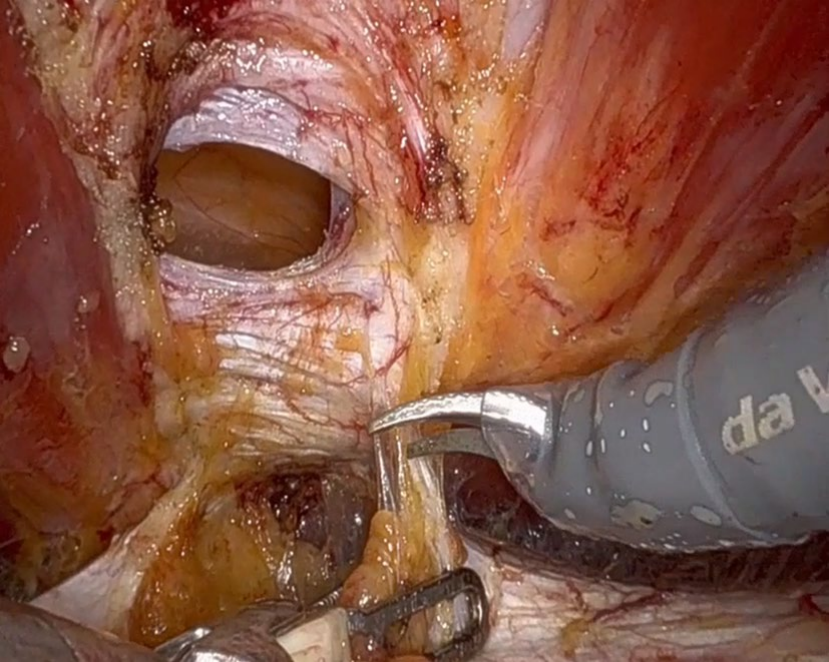
Intraoperative imagine showing the hernia sac reduced and the defect.

**Figure 2:**
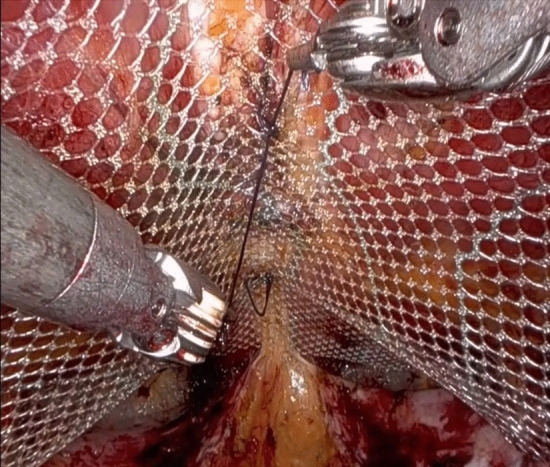
Intraoperative imagine showing the mesh fixed with interrupted absorbable sutures.

All patients are admitted to hospital on the day of surgery and receive a single shot of preoperative antibiotic (cefazolin 2 gr. i.v.) preoperatively. A bladder catheter is routinely placed to leave the bladder empty and minimize the risk of injury. The patients are placed in a supine position, the arms are abducted, the legs straight and joined with the operating table slightly flexed to lower the legs so to avoid any conflict between the robotic arms and the patient’s lower extremities. For the eTEP approach, the first 8 mm da Vinci trocar is inserted two square fingers (about 4 cm) above the pubis in the midline. After the skin incision, the subcutaneous tissue is dissected until the fascia is exposed. After getting through the fascia with the sharp tip of the trocar, the obturator is removed from the trocar and a blunt dissection of the preperitoneal space is performed taking care not to injury the peritoneal layer. Two further 8 mm trocars are inserted under vision laterally to the epigastric vessels and 2–3 cm cranially to the level of the first trocar. The correct angle of lateral trocars placement is optimally figured out through a previous needle insertion. The Da Vinci Xi® patient cart is docked from the right side of the patient. Using a 30° up lens the preperitoneal space is dissected with the fenestrated bipolar forceps on the left (non-dominant hand) and the monopolar curved scissors on the right (dominant) hand. The avascular retrorectus space is dissected from caudally to cranially. On the median line the dissection of the retrorectus planes is completed keeping only the intact peritoneal bridge between the two medial edges of the posterior rectus sheaths. During this so called “crossover”, care must be given not to enter the intraperitoneal cavity or to damage the anterior rectus sheath. Eventual lacerations of the peritoneum or the linea alba are repaired with sutures. Dissecting in a caudal-to cranial-direction, we find the hernia defect and the content is reduced. Laterally, we dissect the entire retrorectus space, being careful not to damage the neurovascular bundles. The intact hernia sack is freed from the fascial defect and the subcutaneous tissue (Fig. 1). The hernia defect is closed with a continuous V-Lock 0 suture. During this maneuver, the carbon dioxide insufflation is reduced to 5–7 mmHg. In case of an umbilical hernia, it is necessary to stitch through the subcutaneous tissue and dermis in order to restore the umbilical shape. In case of diastasis recti, the widened fascia is plicated stitching the anterior rectus aponeurosis with a continuous V-Lock 0 suture. Afterwards, we place a mesh (DynaMesh®-CICAT), whose size depends on the dimension of the created preperitoneal space, which is measured systematically. We fix the mesh with few Vicryl 2-0 interrupted stitches in the midline (Fig. 2). After the undocking and deflation of the pneumoperitoneum, the trocars are removed. We do not close the 8-mm fascia defects. The three skin incisions are finally closed with absorbable Vicryl 3-0 sutures and covered with a spray patch (Video 1).

For the eTAPP approach, the patient is prepared as for the eTEP. The pneumoperitoneum is then established by inserting a Veress needle into the left upper abdomen under the costal margin. Subsequently, three 8 mm trocars are inserted intraperitoneally in the suprapubic area in a straight line. The transverse peritoneal incision, or the peritoneum and the posterior rectus sheath in the case of sublay mesh placement, is made at least 5 cm caudally to the inferior hernia border. The creation of the dissection space, hernia reduction, defect closure, and mesh placement are carried out similarly to eTEP, as described above. Finally, the peritoneum, or both the peritoneum and posterior rectus sheath in the case of retromuscular dissection, are closed with a continuous suture using a 3-0 absorbable barbed suture. Further details were described in a former publication by our research group^7^.

### Statistical analysis

Descriptive statistics were presented as absolute frequencies for categorical variables and mean with standard deviation (SD) for continuous variables. The comparison of categorical variables was carried out with the chi-square test, while for continuous variables the student t-test was used. As no study reported a direct comparison of eTEP and eTAPP with suprapubic approach to treat umbilical and epigastric hernias, no sample size calculation was carried out. A propensity score–matched (PSM) analysis^16^ with 1:1 ratio was carried out according to age, sex, BMI, ASA score, type and dimension of the hernia. MedCalc® Statistical Software version 20.210 was used (MedCalc Software Ltd, Ostend, Belgium; https://www.medcalc.org; 2022).

## Results

During the study period, 53 patients matching inclusion and exclusion criteria were retrieved from our prospectively maintained database. Thirty-two patients were in the eTEP group and 21 in the eTAPP group. Mean age was 59.0 ± 13.9 years, 45 patients (84.9%) were male and mean BMI was 28.0 ± 5.9 kg/m^2^. Details divided into eTEP and eTAPP groups are reported in Table 1. On average, the patients included in this study had few comorbidities (6/53) and small hernia defects (2.5 cm). Most hernias were umbilical (81.1%) and primary (83.0%).

**Table 1:** Patients’ characteristics.

	eTEP N = 32	eTAPP N = 21	p
Age, years (SD)	57.8 (11.9)	60.9 (16.6)	0.423
Gender, male (%)	27 (84.4)	18 (85.7)	0.895
Body mass index, kg/m2 (SD)	27.1 (5.5)	29.5 (6.3)	0.146
ASA score			
1, n (%)	3 (9.4)	4 (19.0)	0.169
2, n (%)	19 (59.4)	7 (33.3)	
3, n (%)	10 (31.3)	10 (47.6)	
Comorbidities, n (%)	3 (9.4)	3 (14.3)	0.585
Pulmonary disease, n (%)	0	0	–
Nicotine use, n (%)	2 (6.2)	0	0.247
Diabetes mellitus, n (%)	1 (3.1)	4 (19.0)	0.055
Immunosuppression, n (%)	0	0	–
Previous abdominal surgery, n (%)	6 (18.8)	3 (14.3)	0.675
Hernia type			
Umbilical, n (%)	27 (84.4)	16 (76.2)	0.758
Epigastric, n (%)	2 (6.2)	2 (9.5)	
Both hernias, n (%)	3 (9.4)	3 (14.3)	
Defect size, cm (SD)	2.5 (0.9)	2.5 (0.8)	0.975
Incisional hernia, n (%)	7 (21.9)	2 (9.5)	0.246

Dichotomous variables are expressed as number with percentage. Continuous variables are expressed as mean with standard deviation (SD).

*ASA* American society of anesthesiology.

Regarding the intraoperative results, we interestingly found that the operative time was shorter for eTEP than eTAPP (106 ± 43 min vs. 126 ± 74 min, p = 0.232). No intraoperative complications occurred, in all cases the hernia defect was closed with absorbable sutures (copolymer of glycolic acid and trimethylene carbonate, V-Lok®) and a transversus muscle release was necessary in only one case (1.9%). The mesh was placed in the retromuscular space in most patients of the eTEP group (87.5%), while in the eTAPP group it was placed in half of the cases in the preperitoneal space and, in the other half, in the retromuscular one. Mesh dimension was similar in both groups and no intraoperative complication occurred.

Postoperatively, only two complications occurred, both in the eTEP group (p = 0.247). The first patient had a postoperative bleeding that required a surgical revision without further complications (grade III Clavien-Dindo). The second one had a seroma in the hernia site and required a percutaneous aspiration (grade III Clavien-Dindo). The latter patient was then followed-up upon resolution after two months. Concerning the length of hospitalization, 10 patients (31.2%) in the eTEP group vs. 6 patients (28.63%) in the eTAPP group were treated as outpatients. Considering the inpatients, the length of stay was 2.6 ± 2.9 days vs. 2.3 ± 1.3 days in the eTEP and eTAPP group respectively. Details were reported in Table 2.

**Table 2:** Intra- and postoperative results.

	eTEP N = 32	eTAPP N = 21	p
Operative time, min (SD)	106 (43)	126 (74)	0.232
Hernia defect closure, n (%)	32 (100)	21 (100)	1.000
Mesh placement			
Preperitoneal, n (%)	4 (12.5)	10 (47.6)	0.005
Retromuscular, n (%)	28 (87.5)	11 (52.4)	
Transversus abdominis release, n (%)	0	1 (4.8%)	0.217
Drainage placement, n (%)	1 (3.1)	3 (9.5)	0.329
Intraoperative complications, n (%)	0	0	–
Mesh size			
Max diameter, cm (SD)	16 (3)	14 (7)	0.298
Min diameter, cm (SD)	14 (2)	13 (4)	0.400
Area, cm^2^ (SD)	214 (42)	211 (245)	0.951
Postoperative complications, n (%)	2 (6.2)	0	0.247
Length of hospital stay			
Outpatients, n (%)	10 (31.2)	6 (28.6)	0.837
Inpatients, days (SD)	2.6 (2.9)	2.3 (1.3)	0.753
Follow-up, months (SD)	11.3 (6.4)	20.5 (9.7)	–

Dichotomous variables are expressed as number with percentage. Continuous variables are expressed as mean with standard deviation (SD).

The length of follow-up was 11.3 ± 6.4 months in the eTEP group vs. 20.5 ± 9.7 months in the eTAPP group and no case of recurrence, chronic pain or significant dysesthesia was recorded.

Finally, a comprehensive PSM analysis was carried out, involving a total of 32 patients, with 16 in each group showing similar baseline characteristics (i.e., age, sex, BMI, ASA score, type, and dimension of the hernia). The results indicated that the operative time still slightly favored eTEP (111 ± 44 vs. 119 ± 82 min, p = 0.727), and in 15 (93.7%) eTEP cases compared to 9 (56.2%) eTAPP cases, the mesh was placed in the retromuscular space (p = 0.016). Other variables, including transversus abdominis release, mesh sizes, postoperative complications, length of hospital stay, and follow-up duration, did not exhibit noteworthy differences from the main analysis.

## Discussion

In our study, we found that the robot-assisted eTEP with suprapubic approach is safe and feasible in treating epigastric and umbilical hernias. Perioperative and follow-up results were similar in the eTEP and eTAPP groups, except for the operative time that was shorter in the eTEP group.

In the treatment of abdominal wall hernia, compared to open procedures, IPOM has significant benefits in terms of shorter hospital stay and recovery^17^. However, visceral complications associated with intraperitoneal mesh placement are still a major concern^2–4^. According to the current literature, in open ventral hernia surgery placing the mesh in sublay technique is associated with a lower rate of complications and recurrence in comparison to other mesh locations^2,18^. Therefore, to combine the benefits of minimally invasive approach and sublay mesh placement, several laparoscopic and, more recently, robot-assisted techniques have been described^19^. The eTEP was first described by Daes in 2012 as a modification of TEP for difficult, large inguinal hernias and obese patients with previous lower abdominal surgery^11^. Technically, the larger operative plane was achieved through a higher optic trocar placement and the division of the line of Douglas. Several authors described how to approach ventral hernias laparoscopically with eTEP reporting good results^12,19–25^. A recent meta-analysis showed that eTEP is associated with shorter hospital stay and lower postoperative pain compared to IPOM in ventral and incisional hernia repair^10^.

Following the widespread adoption of robotic technology in general surgery, several minimally invasive surgeons pointed out how robot-assisted eTEP could overcome the usual ergonomic challenges of laparoscopy. In a recent article by Belyansky et al.^13^, switching from open to laparoscopic to robotic surgery is referred to as “natural evolution of previously established invasive techniques”. Indeed, the robotic magnificent visualization of the operative field and EndoWrist technology-related instruments’ full range of motion allow a highly accurate tissue dissection and reconstruction in narrow spaces^6^.

In our Department of General Surgery, we perform since 2017 robot-assisted abdominal wall surgery, including the treatment of umbilical, inguinal, incisional, ventral, parastomal and flank hernias, as well as of rectus diastasis. In the first years, for the repair of umbilical and epigastric hernia, we performed robotic TAPP procedures with satisfying results^7^. Subsequently, we started using the robot-assisted eTEP with suprapubic approach, until we definitely switched to it.

Compared to what so far described in the literature, our robotic eTEP technique has its own peculiarities. While Belyansky et al.^12^ and Olivier et al.^26^ use a suprapubic approach for upper abdominal wall surgery, Kudsi et al.^27^ prefer a lateral one. Like Belyansky et al. and Olivier et al., our dissection is performed in a caudal-to-cranial direction, which allows the dissection to be started in an unoperated plane. Eventual defects of the peritoneum or rectus sheath layer can be easily closed. We fix the mesh with interrupted stitches, whereas Belyansky et al.^12^ use no fixation and Olivier et al.^26^ prefer a self-gripping mesh. As main difference, while both Belyansky et al. and Olivier et al. perform the initial dissection laparoscopically before docking the robot, we use a fully robotic approach, so that additional laparoscopic material is not required with obvious advantages. Of course, avoiding the initial laparoscopic dissection can get tricky and, eventually, impracticable in case of previous caesarean section, prostatectomy or any pelvic surgery. Nevertheless, based on our experience, such conditions should not be seen as absolute contraindications for our technique. With eTEP, the mesh can be placed in the retromuscular plane and patients undergoing this sort of surgery may be pleased with the aesthetic results of eTEP due to the lack of visible scars.

Of course, the same advantages (dissection in a caudal-to-cranial direction, easy access to the retromuscular space, pleasant cosmetic results) are observed in robotic eTAPP with suprapubic approach. In a recent study comparing transabdominal versus totally extraperitoneal robotic retromuscular techniques for ventral hernia repair, Kudsi et al.^28^ described the latter superior to the former in terms of operative times and surgical site and intraoperative complications. Our results confirmed a shorter operative time in the eTEP group.

In our series, we employed an absorbable suture in both eTEP and eTAPP, but no case of recurrence was recorded during the follow-up. Some studies suggest that absorbable sutures, especially slowly absorbable and long-term absorbable ones, can decrease the incidence of incisional hernias, while other studies claim that nonabsorbable sutures significantly lower the rate of incisional hernia. Allison N et al.^29^ reported that absorbable barbed suture for fascial defect closure in robotic ventral hernia repair was safe, had an acceptable rate of recurrence, and may not be associated with chronic postoperative pain. However, several articles have been published supporting the use of non-absorbable sutures for hernia defect closure. Nguyen D et al.^30^ reported that non-absorbable barbed sutures can be used for rapid and effective primary defect closure in laparoscopic ventral hernia repair. Fox S et al.^31^ found that absorbable suture has a 62% higher rate of surgical site occurrence than permanent suture during ventral hernia repair. A consensus has not been reached in the literature yet, but non-absorbable sutures seem to be more commonly used.

**Figure 3:**
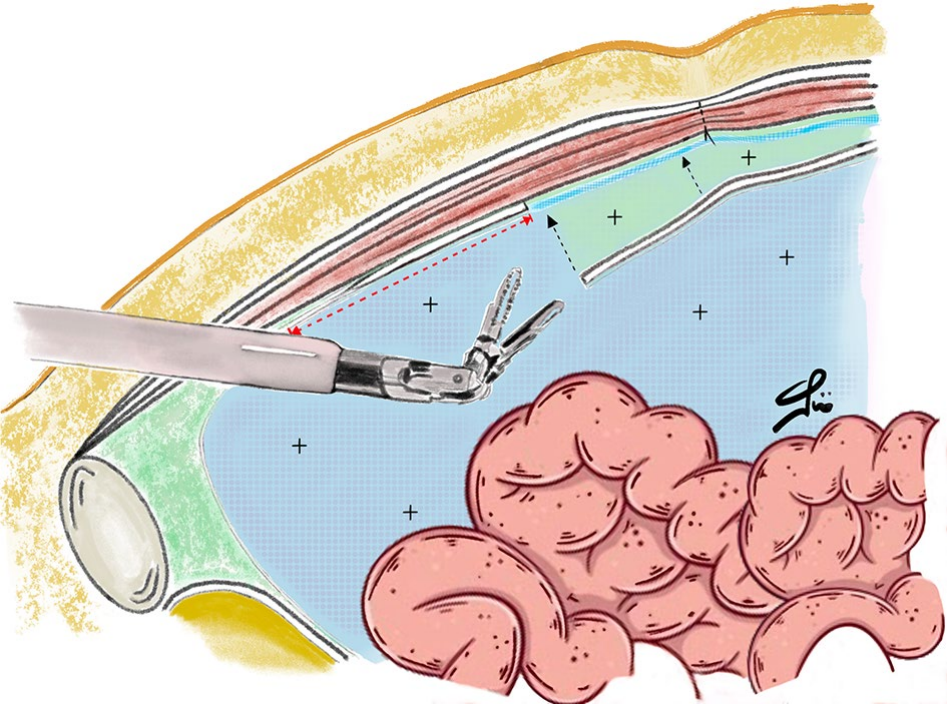
Image showing the intraperitoneal (blue) and the preperitoneal (green) spaces during eTAPP. Note the red dotted line indicating the minimum distance between robotic arms and working area.

**Figure 4:**
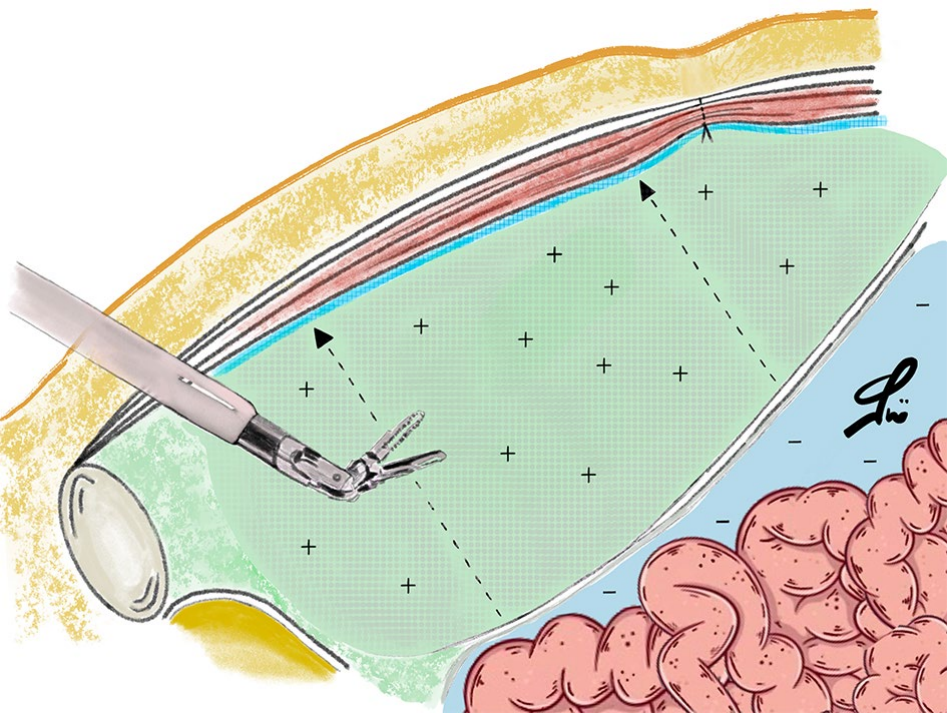
Image showing the preperitoneal (pink) space dissected during eTEP.

Two technical arguments supporting eTEP need to be further discussed. First, in eTEP the integrity of the posterior layers (peritoneum and, above the arcuate line, posterior rectus sheath) is not compromised, whereas the transperitoneal route includes the incision and suture of those components. Then, according to our experience, a wider hernia defect overlap is better achievable with eTEP, specifically when we consider the caudal margin of umbilical defects. To fully understand the latter issue, we should keep in mind that at least 10 cm distance should be maintained between the da Vinci ports and the target anatomy, in order to keep a proper view of the surgical site as well as the range of movements of the robotic instruments. As a consequence, in eTAPP the peritoneum is incised at least 10 cm above the port placement line. Therefore, in eTAPP the mesh can caudally reach this level to overlap the hernia defect. In eTEP, instead, as a peritoneal incision and suture is not needed, the caudal margin of the mesh can be placed 2–3 cm lower inferiorly, with a better mesh area-to-defect ratio in umbilical defects (Figs. 3, 4).

As far as we find eTEP convincing, several issues should be addressed. From a technical point of view, in our eTEP approach, the placement of the first robotic trocar is blind and concerns may rise for the threat of injuring the urinary bladder. However, with careful technique and empty bladder we observed no such complication. Then, the eTEP approach requires the whole dissection of the preperitoneal region below the umbilicus, which could be regarded as excessive in case of epigastric hernias. Such problem is not encountered in eTAPP, as the incision of the peritoneum and posterior layer of the rectus sheath can be carried out at any level. In most patients undergoing eTEP (87.5%), we placed the mesh in the retromuscular space, while, in the eTAPP group, we used in half of the cases the retromuscular space and, in the other half, the preperitoneal one. No specific selection criteria were applied to choose the dissection space but, as a general rule, in patients with a thin peritoneal layer, we tend to avoid the preperitoneal dissection as this may lead to laceration and gapping of the membrane.

Besides the above-mentioned technical issues, we must keep in mind that reaching a safe and competent level of performance in robotic abdominal wall surgery takes time and a realistic learning curve should be considered, which can be variable depending on the single surgeon's experience and training program. Longer operative times, intra- and postoperative complications and high conversion rate have to be taken into account at the beginning of the learning curve^32^. In instances of heightened complexity or a history of prior lower abdominal surgery, achieving suprapubic access may prove challenging. In such scenarios, viable alternative approaches include the lateral placement of the first trocar or the adoption of hybrid techniques, such as the endoscopic mini/less open sublay approach^33^. In addition, despite the growing interest in robotic general surgery, lack of evidence and increase of costs are still a matter of debate among surgeons worldwide^34^.

Also to remember, umbilical and ventral hernias are often considered teaching procedures for surgical residents. Among other considerations, the increasingly frequent use of robotics in abdominal wall surgery will have a certain impact on the proportion of teaching operations among general surgical procedures and, therefore, on the quality of surgical training.

Finally, we must consider the methodological limitations of this study as its retrospective design, although we retrieved the patients from a prospectively maintained database designed for hernia patients. Selection and allocation biases should be carefully taken into account. In addition, the relatively small sample size and differing sizes between the groups may affect the reliability of the analyses and overall generalizability. The applicability of findings may be jeopardized due to the small sample size and imbalance in sizes between groups. Nevertheless, we conducted a PSM analysis to enhance comparability between the groups. Finally, the small sample size does not allow separate analyses for primary and incisional hernias as well as for umbilical and epigastric ones.

In conclusion, our study showed that the eTEP with suprapubic approach was safe and feasible in the treatment of epigastric and umbilical hernias. According to our experience, shorter operative time, integrity of the posterior layers and increased overlap size are the main surgical reasons of switching from eTAPP to eTEP.

### Supplementary Information

Supplementary Video 1.

## Data Availability

The dataset analysed during the current study is available from the corresponding author on request.
